# Supplementary material for: Multi‐dataset identification of innovative feature genes and molecular mechanisms in keratoconus
Source: J Cell Mol Med. 2024 Sep 19;28(18):e70079. doi: 10.1111/jcmm.70079 (PMC11412914; doi:10.1111/jcmm.70079)
Supplement: Supplementary file 2 — Material S2. [file JCMM-28-e70079-s002.zip › 7.GSEA analysis/3.DBNDD1/gsea_report_for_l_1678852837355.html]

Report for l 1678852837355 [GSEA]

| GS  follow link to MSigDB | GS DETAILS | SIZE | ES | NES | NOM p-val | FDR q-val | FWER p-val | RANK AT MAX | LEADING EDGE || 1 | KEGG\_INOSITOL\_PHOSPHATE\_METABOLISM | Details ... | 54 | -0.42 | -1.62 | 0.035 | 1.000 | 0.553 | 11624 | tags=46%, list=33%, signal=69% |
| 2 | KEGG\_ARRHYTHMOGENIC\_RIGHT\_VENTRICULAR\_CARDIOMYOPATHY\_ARVC | Details ... | 74 | -0.51 | -1.60 | 0.014 | 1.000 | 0.622 | 6316 | tags=41%, list=18%, signal=49% |
| 3 | KEGG\_EPITHELIAL\_CELL\_SIGNALING\_IN\_HELICOBACTER\_PYLORI\_INFECTION | Details ... | 66 | -0.45 | -1.59 | 0.053 | 0.869 | 0.632 | 9064 | tags=32%, list=26%, signal=43% |
| 4 | KEGG\_GLYCOSPHINGOLIPID\_BIOSYNTHESIS\_GANGLIO\_SERIES | Details ... | 15 | -0.57 | -1.58 | 0.042 | 0.697 | 0.646 | 2171 | tags=13%, list=6%, signal=14% |
| 5 | KEGG\_THYROID\_CANCER | Details ... | 29 | -0.44 | -1.58 | 0.028 | 0.562 | 0.646 | 7917 | tags=28%, list=23%, signal=36% |
| 6 | KEGG\_DNA\_REPLICATION | Details ... | 36 | -0.46 | -1.51 | 0.024 | 0.785 | 0.776 | 10585 | tags=56%, list=30%, signal=80% |
| 7 | KEGG\_FC\_GAMMA\_R\_MEDIATED\_PHAGOCYTOSIS | Details ... | 92 | -0.38 | -1.51 | 0.056 | 0.704 | 0.787 | 7720 | tags=27%, list=22%, signal=35% |
| 8 | KEGG\_NITROGEN\_METABOLISM | Details ... | 23 | -0.58 | -1.49 | 0.064 | 0.708 | 0.810 | 7691 | tags=43%, list=22%, signal=56% |
| 9 | KEGG\_HUNTINGTONS\_DISEASE | Details ... | 158 | -0.27 | -1.48 | 0.055 | 0.659 | 0.820 | 2949 | tags=6%, list=8%, signal=6% |
| 10 | KEGG\_CELL\_CYCLE | Details ... | 124 | -0.43 | -1.48 | 0.135 | 0.608 | 0.823 | 9073 | tags=35%, list=26%, signal=47% |
| 11 | KEGG\_GLYCOLYSIS\_GLUCONEOGENESIS | Details ... | 61 | -0.41 | -1.47 | 0.049 | 0.577 | 0.832 | 6635 | tags=25%, list=19%, signal=30% |
| 12 | KEGG\_BASAL\_CELL\_CARCINOMA | Details ... | 55 | -0.49 | -1.43 | 0.068 | 0.675 | 0.871 | 3463 | tags=20%, list=10%, signal=22% |
| 13 | KEGG\_MELANOGENESIS | Details ... | 101 | -0.38 | -1.42 | 0.065 | 0.687 | 0.881 | 3740 | tags=16%, list=11%, signal=18% |
| 14 | KEGG\_PROSTATE\_CANCER | Details ... | 89 | -0.34 | -1.41 | 0.071 | 0.675 | 0.887 | 3336 | tags=13%, list=10%, signal=15% |
| 15 | KEGG\_CHEMOKINE\_SIGNALING\_PATHWAY | Details ... | 183 | -0.40 | -1.41 | 0.050 | 0.637 | 0.889 | 8059 | tags=31%, list=23%, signal=40% |
| 16 | KEGG\_GLYCOSAMINOGLYCAN\_BIOSYNTHESIS\_CHONDROITIN\_SULFATE | Details ... | 22 | -0.50 | -1.37 | 0.116 | 0.756 | 0.919 | 7180 | tags=32%, list=21%, signal=40% |
| 17 | KEGG\_HYPERTROPHIC\_CARDIOMYOPATHY\_HCM | Details ... | 83 | -0.39 | -1.34 | 0.087 | 0.829 | 0.944 | 6316 | tags=33%, list=18%, signal=40% |
| 18 | KEGG\_P53\_SIGNALING\_PATHWAY | Details ... | 66 | -0.36 | -1.34 | 0.155 | 0.805 | 0.945 | 8736 | tags=30%, list=25%, signal=40% |
| 19 | KEGG\_INTESTINAL\_IMMUNE\_NETWORK\_FOR\_IGA\_PRODUCTION | Details ... | 48 | -0.50 | -1.33 | 0.089 | 0.789 | 0.949 | 8059 | tags=46%, list=23%, signal=60% |
| 20 | KEGG\_FC\_EPSILON\_RI\_SIGNALING\_PATHWAY | Details ... | 78 | -0.37 | -1.32 | 0.093 | 0.783 | 0.953 | 7716 | tags=31%, list=22%, signal=39% |
| 21 | KEGG\_CYTOKINE\_CYTOKINE\_RECEPTOR\_INTERACTION | Details ... | 256 | -0.43 | -1.31 | 0.098 | 0.784 | 0.958 | 6908 | tags=37%, list=20%, signal=46% |
| 22 | KEGG\_FOCAL\_ADHESION | Details ... | 196 | -0.39 | -1.31 | 0.145 | 0.752 | 0.960 | 6171 | tags=26%, list=18%, signal=31% |
| 23 | KEGG\_GLYCOSAMINOGLYCAN\_BIOSYNTHESIS\_KERATAN\_SULFATE | Details ... | 15 | -0.45 | -1.30 | 0.203 | 0.754 | 0.961 | 6325 | tags=33%, list=18%, signal=41% |
| 24 | KEGG\_TIGHT\_JUNCTION | Details ... | 127 | -0.35 | -1.29 | 0.129 | 0.761 | 0.965 | 6221 | tags=20%, list=18%, signal=25% |
| 25 | KEGG\_ADHERENS\_JUNCTION | Details ... | 68 | -0.33 | -1.29 | 0.229 | 0.734 | 0.965 | 8340 | tags=21%, list=24%, signal=27% |
| 26 | KEGG\_OTHER\_GLYCAN\_DEGRADATION | Details ... | 15 | -0.45 | -1.28 | 0.208 | 0.726 | 0.969 | 644 | tags=7%, list=2%, signal=7% |
| 27 | KEGG\_DILATED\_CARDIOMYOPATHY | Details ... | 90 | -0.35 | -1.28 | 0.113 | 0.714 | 0.971 | 6605 | tags=32%, list=19%, signal=40% |
| 28 | KEGG\_BIOSYNTHESIS\_OF\_UNSATURATED\_FATTY\_ACIDS | Details ... | 19 | -0.37 | -1.28 | 0.146 | 0.690 | 0.971 | 6465 | tags=21%, list=19%, signal=26% |
| 29 | KEGG\_TYROSINE\_METABOLISM | Details ... | 40 | -0.39 | -1.27 | 0.129 | 0.691 | 0.974 | 2863 | tags=20%, list=8%, signal=22% |
| 30 | KEGG\_VASCULAR\_SMOOTH\_MUSCLE\_CONTRACTION | Details ... | 111 | -0.36 | -1.27 | 0.151 | 0.670 | 0.974 | 8400 | tags=29%, list=24%, signal=38% |
| 31 | KEGG\_PATHWAYS\_IN\_CANCER | Details ... | 322 | -0.29 | -1.27 | 0.151 | 0.649 | 0.974 | 8493 | tags=25%, list=24%, signal=32% |
| 32 | KEGG\_ENDOCYTOSIS | Details ... | 179 | -0.24 | -1.27 | 0.186 | 0.636 | 0.974 | 2640 | tags=6%, list=8%, signal=7% |
| 33 | KEGG\_STEROID\_HORMONE\_BIOSYNTHESIS | Details ... | 46 | -0.41 | -1.24 | 0.116 | 0.702 | 0.982 | 3962 | tags=28%, list=11%, signal=32% |
| 34 | KEGG\_PROXIMAL\_TUBULE\_BICARBONATE\_RECLAMATION | Details ... | 23 | -0.40 | -1.23 | 0.165 | 0.707 | 0.985 | 9195 | tags=39%, list=26%, signal=53% |
| 35 | KEGG\_CELL\_ADHESION\_MOLECULES\_CAMS | Details ... | 130 | -0.37 | -1.22 | 0.166 | 0.711 | 0.986 | 6012 | tags=25%, list=17%, signal=31% |
| 36 | KEGG\_RETINOL\_METABOLISM | Details ... | 55 | -0.38 | -1.22 | 0.139 | 0.705 | 0.987 | 3444 | tags=20%, list=10%, signal=22% |
| 37 | KEGG\_SYSTEMIC\_LUPUS\_ERYTHEMATOSUS | Details ... | 56 | -0.50 | -1.21 | 0.270 | 0.701 | 0.988 | 5061 | tags=30%, list=15%, signal=35% |
| 38 | KEGG\_MAPK\_SIGNALING\_PATHWAY | Details ... | 265 | -0.29 | -1.21 | 0.175 | 0.691 | 0.988 | 9454 | tags=30%, list=27%, signal=41% |
| 39 | KEGG\_LYSINE\_DEGRADATION | Details ... | 39 | -0.37 | -1.19 | 0.311 | 0.735 | 0.990 | 12320 | tags=44%, list=35%, signal=67% |
| 40 | KEGG\_CYTOSOLIC\_DNA\_SENSING\_PATHWAY | Details ... | 47 | -0.35 | -1.19 | 0.245 | 0.725 | 0.990 | 8888 | tags=30%, list=26%, signal=40% |
| 41 | KEGG\_BLADDER\_CANCER | Details ... | 41 | -0.41 | -1.18 | 0.309 | 0.711 | 0.990 | 8452 | tags=27%, list=24%, signal=35% |
| 42 | KEGG\_PRION\_DISEASES | Details ... | 35 | -0.41 | -1.18 | 0.229 | 0.703 | 0.991 | 9964 | tags=40%, list=29%, signal=56% |
| 43 | KEGG\_ADIPOCYTOKINE\_SIGNALING\_PATHWAY | Details ... | 66 | -0.33 | -1.17 | 0.278 | 0.710 | 0.991 | 10495 | tags=39%, list=30%, signal=56% |
| 44 | KEGG\_ETHER\_LIPID\_METABOLISM | Details ... | 29 | -0.46 | -1.17 | 0.275 | 0.703 | 0.993 | 7351 | tags=28%, list=21%, signal=35% |
| 45 | KEGG\_METABOLISM\_OF\_XENOBIOTICS\_BY\_CYTOCHROME\_P450 | Details ... | 60 | -0.37 | -1.17 | 0.232 | 0.690 | 0.993 | 7057 | tags=37%, list=20%, signal=46% |
| 46 | KEGG\_CARDIAC\_MUSCLE\_CONTRACTION | Details ... | 72 | -0.31 | -1.17 | 0.172 | 0.685 | 0.993 | 5480 | tags=21%, list=16%, signal=25% |
| 47 | KEGG\_RENAL\_CELL\_CARCINOMA | Details ... | 66 | -0.37 | -1.15 | 0.346 | 0.704 | 0.995 | 7939 | tags=29%, list=23%, signal=37% |
| 48 | KEGG\_SPHINGOLIPID\_METABOLISM | Details ... | 35 | -0.33 | -1.15 | 0.271 | 0.699 | 0.995 | 9134 | tags=31%, list=26%, signal=43% |
| 49 | KEGG\_JAK\_STAT\_SIGNALING\_PATHWAY | Details ... | 151 | -0.34 | -1.15 | 0.276 | 0.693 | 0.995 | 6895 | tags=30%, list=20%, signal=37% |
| 50 | KEGG\_FRUCTOSE\_AND\_MANNOSE\_METABOLISM | Details ... | 32 | -0.40 | -1.14 | 0.337 | 0.691 | 0.995 | 10652 | tags=41%, list=31%, signal=59% |
| 51 | KEGG\_GLUTATHIONE\_METABOLISM |  | 49 | -0.34 | -1.13 | 0.316 | 0.714 | 0.998 | 8044 | tags=24%, list=23%, signal=32% |
| 52 | KEGG\_LONG\_TERM\_DEPRESSION |  | 67 | -0.34 | -1.13 | 0.284 | 0.701 | 0.998 | 8190 | tags=27%, list=24%, signal=35% |
| 53 | KEGG\_AXON\_GUIDANCE |  | 128 | -0.29 | -1.12 | 0.288 | 0.704 | 0.999 | 9089 | tags=31%, list=26%, signal=42% |
| 54 | KEGG\_SMALL\_CELL\_LUNG\_CANCER |  | 84 | -0.29 | -1.11 | 0.341 | 0.730 | 0.999 | 9303 | tags=33%, list=27%, signal=45% |
| 55 | KEGG\_REGULATION\_OF\_ACTIN\_CYTOSKELETON |  | 210 | -0.26 | -1.11 | 0.290 | 0.720 | 0.999 | 6125 | tags=17%, list=18%, signal=21% |
| 56 | KEGG\_FATTY\_ACID\_METABOLISM |  | 42 | -0.33 | -1.10 | 0.333 | 0.719 | 0.999 | 5233 | tags=19%, list=15%, signal=22% |
| 57 | KEGG\_DRUG\_METABOLISM\_CYTOCHROME\_P450 |  | 62 | -0.34 | -1.10 | 0.311 | 0.709 | 0.999 | 7057 | tags=32%, list=20%, signal=40% |
| 58 | KEGG\_GRAFT\_VERSUS\_HOST\_DISEASE |  | 39 | -0.44 | -1.10 | 0.370 | 0.703 | 0.999 | 7782 | tags=33%, list=22%, signal=43% |
| 59 | KEGG\_ECM\_RECEPTOR\_INTERACTION |  | 84 | -0.35 | -1.10 | 0.352 | 0.693 | 0.999 | 9258 | tags=38%, list=27%, signal=52% |
| 60 | KEGG\_LEISHMANIA\_INFECTION |  | 71 | -0.39 | -1.09 | 0.370 | 0.694 | 0.999 | 9721 | tags=45%, list=28%, signal=62% |
| 61 | KEGG\_LEUKOCYTE\_TRANSENDOTHELIAL\_MIGRATION |  | 114 | -0.29 | -1.09 | 0.300 | 0.692 | 0.999 | 5871 | tags=19%, list=17%, signal=23% |
| 62 | KEGG\_NATURAL\_KILLER\_CELL\_MEDIATED\_CYTOTOXICITY |  | 125 | -0.29 | -1.07 | 0.324 | 0.724 | 1.000 | 4520 | tags=17%, list=13%, signal=19% |
| 63 | KEGG\_WNT\_SIGNALING\_PATHWAY |  | 149 | -0.26 | -1.07 | 0.324 | 0.719 | 1.000 | 3463 | tags=10%, list=10%, signal=11% |
| 64 | KEGG\_HOMOLOGOUS\_RECOMBINATION |  | 26 | -0.33 | -1.07 | 0.407 | 0.722 | 1.000 | 10182 | tags=38%, list=29%, signal=54% |
| 65 | KEGG\_LINOLEIC\_ACID\_METABOLISM |  | 28 | -0.40 | -1.07 | 0.368 | 0.711 | 1.000 | 2982 | tags=18%, list=9%, signal=20% |
| 66 | KEGG\_ASTHMA |  | 29 | -0.50 | -1.06 | 0.434 | 0.709 | 1.000 | 3516 | tags=28%, list=10%, signal=31% |
| 67 | KEGG\_RIG\_I\_LIKE\_RECEPTOR\_SIGNALING\_PATHWAY |  | 64 | -0.28 | -1.06 | 0.378 | 0.705 | 1.000 | 4237 | tags=13%, list=12%, signal=14% |
| 68 | KEGG\_ALDOSTERONE\_REGULATED\_SODIUM\_REABSORPTION |  | 42 | -0.32 | -1.06 | 0.334 | 0.700 | 1.000 | 5833 | tags=24%, list=17%, signal=29% |
| 69 | KEGG\_ALLOGRAFT\_REJECTION |  | 37 | -0.44 | -1.06 | 0.404 | 0.692 | 1.000 | 7782 | tags=38%, list=22%, signal=49% |
| 70 | KEGG\_AMYOTROPHIC\_LATERAL\_SCLEROSIS\_ALS |  | 53 | -0.30 | -1.05 | 0.383 | 0.693 | 1.000 | 9351 | tags=32%, list=27%, signal=44% |
| 71 | KEGG\_PURINE\_METABOLISM |  | 151 | -0.24 | -1.05 | 0.417 | 0.691 | 1.000 | 8190 | tags=19%, list=24%, signal=25% |
| 72 | KEGG\_LYSOSOME |  | 117 | -0.20 | -1.04 | 0.403 | 0.695 | 1.000 | 1727 | tags=4%, list=5%, signal=4% |
| 73 | KEGG\_ALZHEIMERS\_DISEASE |  | 144 | -0.20 | -1.04 | 0.431 | 0.688 | 1.000 | 8217 | tags=13%, list=24%, signal=16% |
| 74 | KEGG\_TGF\_BETA\_SIGNALING\_PATHWAY |  | 85 | -0.31 | -1.04 | 0.423 | 0.695 | 1.000 | 8684 | tags=31%, list=25%, signal=41% |
| 75 | KEGG\_T\_CELL\_RECEPTOR\_SIGNALING\_PATHWAY |  | 107 | -0.29 | -1.03 | 0.425 | 0.693 | 1.000 | 7996 | tags=27%, list=23%, signal=35% |
| 76 | KEGG\_GLIOMA |  | 65 | -0.24 | -1.03 | 0.409 | 0.695 | 1.000 | 3006 | tags=11%, list=9%, signal=12% |
| 77 | KEGG\_GLYCOSPHINGOLIPID\_BIOSYNTHESIS\_LACTO\_AND\_NEOLACTO\_SERIES |  | 26 | -0.35 | -1.03 | 0.439 | 0.690 | 1.000 | 2178 | tags=12%, list=6%, signal=12% |
| 78 | KEGG\_HEDGEHOG\_SIGNALING\_PATHWAY |  | 55 | -0.32 | -1.02 | 0.430 | 0.683 | 1.000 | 3463 | tags=15%, list=10%, signal=16% |
| 79 | KEGG\_REGULATION\_OF\_AUTOPHAGY |  | 30 | -0.33 | -1.02 | 0.407 | 0.696 | 1.000 | 4237 | tags=17%, list=12%, signal=19% |
| 80 | KEGG\_ANTIGEN\_PROCESSING\_AND\_PRESENTATION |  | 79 | -0.31 | -1.01 | 0.438 | 0.689 | 1.000 | 4237 | tags=11%, list=12%, signal=13% |
| 81 | KEGG\_TOLL\_LIKE\_RECEPTOR\_SIGNALING\_PATHWAY |  | 96 | -0.29 | -1.01 | 0.423 | 0.687 | 1.000 | 7819 | tags=26%, list=23%, signal=34% |
| 82 | KEGG\_NUCLEOTIDE\_EXCISION\_REPAIR |  | 43 | -0.24 | -1.01 | 0.462 | 0.682 | 1.000 | 13192 | tags=44%, list=38%, signal=71% |
| 83 | KEGG\_MISMATCH\_REPAIR |  | 23 | -0.29 | -1.01 | 0.466 | 0.682 | 1.000 | 11592 | tags=48%, list=33%, signal=72% |
| 84 | KEGG\_TYPE\_I\_DIABETES\_MELLITUS |  | 43 | -0.38 | -1.00 | 0.473 | 0.686 | 1.000 | 1841 | tags=12%, list=5%, signal=12% |
| 85 | KEGG\_COMPLEMENT\_AND\_COAGULATION\_CASCADES |  | 69 | -0.32 | -1.00 | 0.439 | 0.692 | 1.000 | 5219 | tags=28%, list=15%, signal=32% |
| 86 | KEGG\_INSULIN\_SIGNALING\_PATHWAY |  | 136 | -0.23 | -0.99 | 0.482 | 0.691 | 1.000 | 8814 | tags=23%, list=25%, signal=30% |
| 87 | KEGG\_VIRAL\_MYOCARDITIS |  | 70 | -0.31 | -0.99 | 0.466 | 0.694 | 1.000 | 5585 | tags=20%, list=16%, signal=24% |
| 88 | KEGG\_VEGF\_SIGNALING\_PATHWAY |  | 75 | -0.26 | -0.98 | 0.464 | 0.708 | 1.000 | 6415 | tags=17%, list=18%, signal=21% |
| 89 | KEGG\_PYRUVATE\_METABOLISM |  | 40 | -0.25 | -0.97 | 0.514 | 0.721 | 1.000 | 6593 | tags=15%, list=19%, signal=18% |
| 90 | KEGG\_MATURITY\_ONSET\_DIABETES\_OF\_THE\_YOUNG |  | 25 | -0.37 | -0.96 | 0.477 | 0.724 | 1.000 | 7682 | tags=44%, list=22%, signal=56% |
| 91 | KEGG\_ALPHA\_LINOLENIC\_ACID\_METABOLISM |  | 18 | -0.41 | -0.96 | 0.534 | 0.726 | 1.000 | 6123 | tags=22%, list=18%, signal=27% |
| 92 | KEGG\_TRYPTOPHAN\_METABOLISM |  | 39 | -0.29 | -0.95 | 0.530 | 0.727 | 1.000 | 7057 | tags=26%, list=20%, signal=32% |
| 93 | KEGG\_AUTOIMMUNE\_THYROID\_DISEASE |  | 48 | -0.35 | -0.95 | 0.523 | 0.727 | 1.000 | 4237 | tags=21%, list=12%, signal=24% |
| 94 | KEGG\_BETA\_ALANINE\_METABOLISM |  | 22 | -0.34 | -0.94 | 0.563 | 0.734 | 1.000 | 6627 | tags=18%, list=19%, signal=22% |
| 95 | KEGG\_PATHOGENIC\_ESCHERICHIA\_COLI\_INFECTION |  | 54 | -0.21 | -0.94 | 0.548 | 0.731 | 1.000 | 3555 | tags=7%, list=10%, signal=8% |
| 96 | KEGG\_GAP\_JUNCTION |  | 86 | -0.21 | -0.94 | 0.600 | 0.726 | 1.000 | 6605 | tags=16%, list=19%, signal=20% |
| 97 | KEGG\_TASTE\_TRANSDUCTION |  | 52 | -0.32 | -0.94 | 0.599 | 0.725 | 1.000 | 1286 | tags=12%, list=4%, signal=12% |
| 98 | KEGG\_ONE\_CARBON\_POOL\_BY\_FOLATE |  | 17 | -0.34 | -0.94 | 0.538 | 0.719 | 1.000 | 8036 | tags=24%, list=23%, signal=31% |
| 99 | KEGG\_PHOSPHATIDYLINOSITOL\_SIGNALING\_SYSTEM |  | 76 | -0.22 | -0.93 | 0.528 | 0.719 | 1.000 | 5789 | tags=13%, list=17%, signal=16% |
| 100 | KEGG\_CHRONIC\_MYELOID\_LEUKEMIA |  | 73 | -0.23 | -0.93 | 0.523 | 0.712 | 1.000 | 8340 | tags=25%, list=24%, signal=32% |
| 101 | KEGG\_PRIMARY\_BILE\_ACID\_BIOSYNTHESIS |  | 16 | -0.41 | -0.93 | 0.563 | 0.706 | 1.000 | 8806 | tags=44%, list=25%, signal=59% |
| 102 | KEGG\_GALACTOSE\_METABOLISM |  | 25 | -0.31 | -0.93 | 0.566 | 0.707 | 1.000 | 8698 | tags=28%, list=25%, signal=37% |
| 103 | KEGG\_MTOR\_SIGNALING\_PATHWAY |  | 51 | -0.27 | -0.92 | 0.546 | 0.715 | 1.000 | 6278 | tags=18%, list=18%, signal=22% |
| 104 | KEGG\_B\_CELL\_RECEPTOR\_SIGNALING\_PATHWAY |  | 75 | -0.22 | -0.92 | 0.622 | 0.717 | 1.000 | 8343 | tags=25%, list=24%, signal=33% |
| 105 | KEGG\_NEUROTROPHIN\_SIGNALING\_PATHWAY |  | 125 | -0.19 | -0.92 | 0.556 | 0.711 | 1.000 | 9489 | tags=23%, list=27%, signal=32% |
| 106 | KEGG\_MELANOMA |  | 71 | -0.27 | -0.92 | 0.579 | 0.705 | 1.000 | 3006 | tags=11%, list=9%, signal=12% |
| 107 | KEGG\_ACUTE\_MYELOID\_LEUKEMIA |  | 57 | -0.22 | -0.91 | 0.593 | 0.707 | 1.000 | 12436 | tags=46%, list=36%, signal=71% |
| 108 | KEGG\_NOD\_LIKE\_RECEPTOR\_SIGNALING\_PATHWAY |  | 61 | -0.28 | -0.91 | 0.518 | 0.706 | 1.000 | 10711 | tags=38%, list=31%, signal=54% |
| 109 | KEGG\_ARACHIDONIC\_ACID\_METABOLISM |  | 57 | -0.27 | -0.90 | 0.627 | 0.704 | 1.000 | 6123 | tags=23%, list=18%, signal=28% |
| 110 | KEGG\_VIBRIO\_CHOLERAE\_INFECTION |  | 51 | -0.22 | -0.90 | 0.588 | 0.699 | 1.000 | 1000 | tags=4%, list=3%, signal=4% |
| 111 | KEGG\_NOTCH\_SIGNALING\_PATHWAY |  | 47 | -0.24 | -0.90 | 0.573 | 0.707 | 1.000 | 11210 | tags=32%, list=32%, signal=47% |
| 112 | KEGG\_ERBB\_SIGNALING\_PATHWAY |  | 86 | -0.20 | -0.89 | 0.597 | 0.710 | 1.000 | 7857 | tags=20%, list=23%, signal=25% |
| 113 | KEGG\_TYPE\_II\_DIABETES\_MELLITUS |  | 47 | -0.29 | -0.88 | 0.614 | 0.722 | 1.000 | 8814 | tags=36%, list=25%, signal=48% |
| 114 | KEGG\_HISTIDINE\_METABOLISM |  | 28 | -0.33 | -0.88 | 0.617 | 0.726 | 1.000 | 6635 | tags=29%, list=19%, signal=35% |
| 115 | KEGG\_RENIN\_ANGIOTENSIN\_SYSTEM |  | 17 | -0.38 | -0.87 | 0.630 | 0.729 | 1.000 | 6204 | tags=24%, list=18%, signal=29% |
| 116 | KEGG\_LONG\_TERM\_POTENTIATION |  | 70 | -0.21 | -0.84 | 0.702 | 0.776 | 1.000 | 8000 | tags=20%, list=23%, signal=26% |
| 117 | KEGG\_STEROID\_BIOSYNTHESIS |  | 17 | -0.27 | -0.84 | 0.638 | 0.771 | 1.000 | 2796 | tags=12%, list=8%, signal=13% |
| 118 | KEGG\_CALCIUM\_SIGNALING\_PATHWAY |  | 177 | -0.22 | -0.84 | 0.835 | 0.777 | 1.000 | 4073 | tags=15%, list=12%, signal=17% |
| 119 | KEGG\_O\_GLYCAN\_BIOSYNTHESIS |  | 29 | -0.29 | -0.83 | 0.683 | 0.787 | 1.000 | 2171 | tags=14%, list=6%, signal=15% |
| 120 | KEGG\_HEMATOPOIETIC\_CELL\_LINEAGE |  | 87 | -0.27 | -0.83 | 0.776 | 0.781 | 1.000 | 7268 | tags=32%, list=21%, signal=41% |
| 121 | KEGG\_AMINO\_SUGAR\_AND\_NUCLEOTIDE\_SUGAR\_METABOLISM |  | 42 | -0.23 | -0.82 | 0.632 | 0.783 | 1.000 | 10501 | tags=29%, list=30%, signal=41% |
| 122 | KEGG\_UBIQUITIN\_MEDIATED\_PROTEOLYSIS |  | 130 | -0.14 | -0.80 | 0.721 | 0.815 | 1.000 | 28822 | tags=99%, list=83%, signal=581% |
| 123 | KEGG\_PENTOSE\_PHOSPHATE\_PATHWAY |  | 27 | -0.21 | -0.80 | 0.793 | 0.809 | 1.000 | 6232 | tags=15%, list=18%, signal=18% |
| 124 | KEGG\_PROGESTERONE\_MEDIATED\_OOCYTE\_MATURATION |  | 85 | -0.19 | -0.78 | 0.739 | 0.838 | 1.000 | 9073 | tags=25%, list=26%, signal=33% |
| 125 | KEGG\_PYRIMIDINE\_METABOLISM |  | 95 | -0.18 | -0.77 | 0.687 | 0.857 | 1.000 | 13079 | tags=34%, list=38%, signal=54% |
| 126 | KEGG\_PROTEASOME |  | 43 | -0.22 | -0.77 | 0.801 | 0.852 | 1.000 | 1013 | tags=2%, list=3%, signal=2% |
| 127 | KEGG\_DORSO\_VENTRAL\_AXIS\_FORMATION |  | 24 | -0.24 | -0.76 | 0.744 | 0.853 | 1.000 | 12689 | tags=50%, list=37%, signal=79% |
| 128 | KEGG\_BASE\_EXCISION\_REPAIR |  | 33 | -0.25 | -0.76 | 0.682 | 0.852 | 1.000 | 13618 | tags=48%, list=39%, signal=80% |
| 129 | KEGG\_GNRH\_SIGNALING\_PATHWAY |  | 100 | -0.17 | -0.75 | 0.906 | 0.854 | 1.000 | 6605 | tags=13%, list=19%, signal=16% |
| 130 | KEGG\_PPAR\_SIGNALING\_PATHWAY |  | 69 | -0.22 | -0.73 | 0.884 | 0.876 | 1.000 | 7605 | tags=25%, list=22%, signal=31% |
| 131 | KEGG\_VASOPRESSIN\_REGULATED\_WATER\_REABSORPTION |  | 44 | -0.19 | -0.69 | 0.839 | 0.920 | 1.000 | 170 | tags=2%, list=0%, signal=2% |
| 132 | KEGG\_PANTOTHENATE\_AND\_COA\_BIOSYNTHESIS |  | 16 | -0.26 | -0.69 | 0.848 | 0.915 | 1.000 | 10998 | tags=38%, list=32%, signal=55% |
| 133 | KEGG\_CITRATE\_CYCLE\_TCA\_CYCLE |  | 30 | -0.17 | -0.69 | 0.874 | 0.909 | 1.000 | 28679 | tags=100%, list=83%, signal=574% |
| 134 | KEGG\_SNARE\_INTERACTIONS\_IN\_VESICULAR\_TRANSPORT |  | 38 | -0.20 | -0.68 | 0.837 | 0.919 | 1.000 | 6372 | tags=11%, list=18%, signal=13% |
| 135 | KEGG\_BASAL\_TRANSCRIPTION\_FACTORS |  | 33 | -0.16 | -0.67 | 0.818 | 0.923 | 1.000 | 13991 | tags=45%, list=40%, signal=76% |
| 136 | KEGG\_ALANINE\_ASPARTATE\_AND\_GLUTAMATE\_METABOLISM |  | 30 | -0.20 | -0.66 | 0.951 | 0.923 | 1.000 | 9702 | tags=30%, list=28%, signal=42% |
| 137 | KEGG\_N\_GLYCAN\_BIOSYNTHESIS |  | 46 | -0.20 | -0.63 | 0.797 | 0.941 | 1.000 | 27966 | tags=100%, list=81%, signal=513% |
| 138 | KEGG\_GLYCEROPHOSPHOLIPID\_METABOLISM |  | 70 | -0.15 | -0.58 | 0.992 | 0.970 | 1.000 | 6123 | tags=11%, list=18%, signal=14% |
| 139 | KEGG\_PROPANOATE\_METABOLISM |  | 31 | -0.17 | -0.53 | 0.943 | 0.986 | 1.000 | 6593 | tags=10%, list=19%, signal=12% |
| 140 | KEGG\_SELENOAMINO\_ACID\_METABOLISM |  | 24 | -0.15 | -0.45 | 0.996 | 0.995 | 1.000 | 12003 | tags=38%, list=35%, signal=57% |
Table: Gene sets enriched in phenotype **l (8 samples) **[plain text format]****
